# Physiochemical and Nutritional Characteristics of Ready-to-Use Therapeutic Food Prepared Using Bambara Groundnut-*Moringa oleifera* Leaf Protein Complex

**DOI:** 10.3390/foods11121680

**Published:** 2022-06-08

**Authors:** Olawumi Oluwakemi Adewumi, Joseline Veronica Felix-Minnaar, Victoria Adaora Jideani

**Affiliations:** Department of Food Science and Technology, Cape Peninsula University of Technology, Bellville 7555, South Africa; kemitale@yahoo.com (O.O.A.); felixminnaarj@cput.ac.za (J.V.F.-M.)

**Keywords:** Bambara groundnut-*Moringa oleifera* leaf protein complex, ready-to-use therapeutic food, amino acid, nutritional, physicochemical

## Abstract

The utilisation of local raw material in the production of ready-to-use therapeutic food (RUTF) is worthy of exploration for the replacement of full-fat milk, peanut butter, mineral and vitamin mix used in the standard formulation. The objective of this study was to produce snack bars that will meet the protein requirement set by World Health Organisation (WHO) for RUTF (13–16% by weight) using the Bambara groundnut-*Moringa oleifera* leaf protein complex (BAMOLP). The BAMOnut snack bars were simulated using the mixture preparation procedure in Superpro Designer to determine different proportions of BAMOLP, *Moringa oleifera* leaf powder, egusi, oats, and millet. Three bars formulated were; BAMOnut-OB3 (BAMOnut Bar enriched with oats and 3% BAMOLP), BAMOnut-MB2 (BAMOnut Bar enriched with millet and 2% BAMOLP), and BAMOnut-OMB5 (BAMOnut Bar enriched with oats, millets, and 5% BAMOLP). The snack bars were assessed for physical, nutritional, proximate and bench-top sensory properties. BAMOnut-OB3 was firmer and less crumbly, with a larger particle size. BAMOnut-OB3 had the lowest water activity, lightest colour and the best amino acid profile. The moisture (4.9%), protein (14.1%), fat (19.3%), carbohydrate (59.7%), and energy (468.6 Kcal/100 g) of BAMOnut-OB3, compare favourably with the requirements for RUTF (2.5% moisture, 13–16% protein, 26–36% fat, 41–58% carbohydrate, and 520–550 Kcal/100 g energy). Local raw materials can be successfully used in the production of RUTF.

## 1. Introduction

Bambara groundnut-*Moringa oleifera* leaf protein complex (BAMOLP) is an innovative product [1]. BAMOLP is a combination of Bambara groundnut protein isolate and *Moringa oleifera* leaf protein isolate. It is a potential functional food ingredient and a sustainable raw material for the food industry due to its nutritional profile as reported by [1]. BAMOLP is a good source of protein and is rich in essential amino acid. It is higher in methionine than whey, pea, and soy protein, as reported by [2], and is higher in phenylalanine compared with whey, pea, brown rice, soy, hemp and wheat protein, as reported by [2,3]. It can therefore be used as an alternative in applications where these proteins are required. The amino acid content of BAMOLP is higher in threonine, phenylalanine lysine, and leucine than the Food and Agricultural Organization/World Health Organization (FAO/WHO) reference pattern [1,4]. Due to the appreciable content of lysine in BAMOLP, it can be used as a supplementary protein for cereals, which are deficient in lysine.

Ready-to-use therapeutic foods (RUTF) have been confirmed effective in the management of severe acute malnutrition, but some ingredients needed for their production are not available locally. Plumpy’Nut, Imunut, and BP-100 are the commercial forms of RUTF. Bambara groundnut (BGN) and *Moringa oliefera* leaf powder (MOLP) are nutrient-dense foods with quality protein that can be used as local raw material in the production of RUTF. Both are rich in amino acids, minerals, and vitamins. Nevertheless, little is known about their food value application, especially in the context of malnutrition. Complementing BGN protein with MOLP will provide a complete protein to compete with other ingredients used in the production of RUTF, and will meet the protein requirement (10–12% of the total energy) for RUTF set by WHO.

Ingredients for standard RUTF are full-fat milk, peanut, vegetable oil, sugar, and mineral and vitamin mix. Commonly used ingredients for the alternative RUTF are a legume (almond, groundnut, lentil, and soybean), cereal/grain (maize and oats), milk (whey protein concentrate), oil (canola, palm, sunflower, soybean, and coconut), sugar and cocoa powder. This study aimed at the development of a snack bar that will meet RUTF requirements, through fortification with Bambara groundnut-*Moringa oleifera* leaf protein complex (BAMOLP). The BAMOLP should increase protein content, and served as a replacement for full milk powder. *Moringa oleifera* leaf powder (MOLP) was used for the vitamin and mineral mix, egusi melon (*Citrullus lanatus sub-Mucosospermus*) as a replacement for peanut which is often exposed to aflatoxin, and millet and oat were used as an energy source [5,6].

## 2. Materials and Methods

### 2.1. Material

The BGN seeds were purchased from Triotrade Johannesburg, South Africa; MO leaf from *Moringa* Africa, Johannesburg; millet from AGT Foods Africa, Cape Town; egusi came from a local seed store in Cape Town. Chemicals were purchased from Merck Pty Ltd., Cape Town, South Africa. Food ingredients were purchased from Bidvest FoodService, Cape Town, South Africa.

### 2.2. Pre-Processing of Millet and Melon Seed (Egusi)

The millet was dehulled by boiling, followed by treatment with calcium hydroxide to remove the seed coat. Calcium hydroxide at 5 g was added to 1 L boiling water, after which 1 kg millet was added to the solution. The millet in the solution was stirred with a spoon. It was then drained, and poured into 1% citric acid. It was again rinsed, washed, drained, and dried at 50 °C for 4 h, it was then crushed and ground in a mortar and pestle to reduce the particle size.

The egusi was sorted to remove dirt, after which it was washed, drained, and dried in the dehydrator at 50 °C for 6 h and roasted at 180 °C for 6–7 min. It was afterwards milled in a Kenwood blender at setting 3, and packed in a zip-locked bag, and kept in the fridge at 4 °C until further use.

### 2.3. Simulation of Ingredient Proportions to Meet the Ready-to-Use Therapeutic Requirement Using Superpro Designer

The proximate compositions of BAMOLP, egusi, MOLP, and oats used in the simulation are outlined in Table 1. The cereals used as the source of energy for the bars were millet and oats. Egusi was chosen as the oilseed for the base to replace the commonly used peanut butter. This study aimed to produce three bars consisting of BAMOLP + MOLP + oats + egusi, BAMOLP + MOLP + millet + egusi, and BAMOLP + MOLP + millet + oats + egusi. The objective in each case was to obtain bars with fat at 45–60% total energy or 26–36% by weight, protein at 10–12% total energy or 13–16% by weight, and moisture 2.5% max.

**Figure 1:**
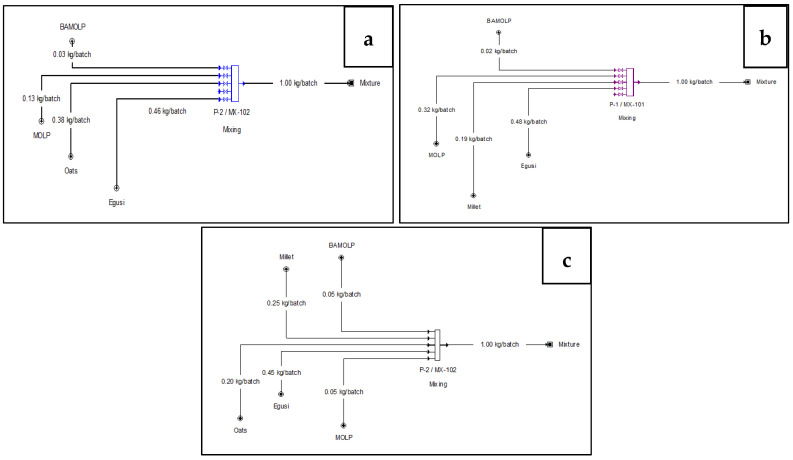
Process flow diagram of Bambara groundnut-*Moringa oleifera* snack bar enriched with Bambara groundnut-Moringa oleifera leaf protein (BAMOLP) at low, medium, and high concentrations, (**a**) oats plus 3% BAMOLP, (**b**) millet plus 2% BAMOLP, (**c**) oats, millet, and 5% BAMOLP.

The mixture preparation procedure in SuperPro designer (Version 9) was used to obtain the appropriate proportion of components to meet the optimisation goal. The mixture preparation procedure simulates an intelligent mixer that automatically adjusts the flow of its input streams to meet the objective goal. The objective goal for each bar is detailed in Table 2. The simulation results are depicted in Figure 1.

### 2.4. Production of Bambara Groundnut and Moringa oleifera Snack Bar

Three varieties of Bambara groundnut and *Moringa oleifera* (BAMOnut) snack bar were produced. The main constituents of the bars were determined from the simulation as listed in Table 3. Other ingredients were added to each batch, namely, 2 g cocoa powder, 30 g icing sugar, 15 g canola oil, and 45 g honey. First, the dry ingredients were weighed into the Kenwood blender, and mixed on speed setting 1 for 2 min, then speed setting 3 for 3 min. It was then transferred into a mixing bowl, the wet ingredients were added, and the contents were mixed until well combined. A 250 g batch was produced, the mixture was taken out of the bowl, and placed in a pre-lined baking pan. The mixture was baked in a pre-heated oven at 160 °C for 30 min and cooled at room temperature, thereafter it was cut into a rectangular shape.

### 2.5. Physical Properties of BAMOnut

#### 2.5.1. Morphology (Characterisation) of BAMOnut Bars

1 Scanning electron microscopy-energy dispersive X-ray spectrometry (SEM-EDX) analysis of BAMOnut snack bars.

Scanning electron microscope (SEM) analysis was carried out using Zeiss MERLIN (Carl Zeiss Microscopy, Jena, Germany). Samples were placed on carbon double-sided adhesive tape mounted on Aluminum SEM stubs. They were observed using a scanning electron microscope operated at an acceleration voltage of 20 kV.

2 Scanning transmission electron microscopy (STEM)

Basic STEM preparation was carried out for the BAMOnut snack bar samples. Pieces of the samples were dispersed in ethanol, and then sonicated for an hour. For each sample a drop of the solution was placed in a Petri dish and a FORMVAR-coated 200 Mesh Cu TEM grid was placed on the droplet. The Grid was then rinsed in distilled water and placed on a droplet of UA zero (uranium free urinal acetate).

#### 2.5.2. The Water Activity of BAMOnut Bars

Water activity (a_w_) of the BAMOnut bars (oat with *Moringa*, millet with *Moringa oleifera* *leaf powder*, and oat and millet with *Moringa*) was measured using a Novasina Ms 1 Set a_w_ meter with a cell protection filter. The measurement cell was calibrated with salt humidity standards of 53, 75, and 90%. The three variations of BAMOnut were transferred individually into the sample container. The sample container was placed inside the Novasina analyser and the cell measuring protection filter was immediately closed. The water activity reading was observed for stability and the values were recorded [11]. The test was carried out in triplicate.

#### 2.5.3. Colour Measurement

The colours of the BAMOnut snack bars were measured using a Spectrophotometer (Model CM-5.45°/0° standard, Konica Minolta Sensing, Osaka, Japan), set at standard observer 10° and D65 [12]. The instrument was calibrated using a black tile (L* = 5.49, a* = 7.08, b* = 4.66) and a white tile (L* = 93.41, a* = −1.18, b* = 0.75), followed by zero calibration. The L* coordinate refers to lightness, 100 represents white and 0 represents black, a* is (chromaticity coordinate +a* = red and −a* = green), b* is (chromaticity coordinate +b* = yellow and −b* = blue), C* = Chroma, h = hue angle (0° = +a*, 90° = +b*, 180° = −a* and 270° = −b*). The BAMOnut bars were crushed using a mortar and pestle. The samples were placed in a sample-dish (30 mm diameter) and reflectance was measured for L*a*b* and L*C*h* colour space systems. Measurements were taken in triplicate for each sample (one reading = average of three readings per rotated position). The total colour difference (ΔE) was estimated using Equation (1).

(1) $$\Delta E=\sqrt{{\left({\Delta L}^{*}\right)}^{2}+{\left({\Delta a}^{*}\right)}^{2}+{\left({\Delta b}^{*}\right)}^{2}}$$

#### 2.5.4. Textural Properties of BAMOnut Bars

BAMOnut Bars were analysed for hardness, springiness, cohesiveness, and chewiness, using the Instron Model 3322 [13]. For the texture profile analysis (TPA), the BAMOnut bar was cut into 4 cm length × 1 cm height with a knife and put on the support plate, then compressed with a compression anvil with pre-test conditions set at 2.00 mm/s, test at 2.0 mm/s, and post-test at 2.00 mm/s [14]. The BAMOnut bars were cut into 2.5 cm length by 1 cm height for the puncture test and the pre-test speed was set at 1 mm/s, the probe used was 5 mm in diameter. The cutting test was performed using the Warner-Bratzler Shear Force (WBSF) method [15]. The WBSF blade was mounted on the Instron device, the crosshead speed for the pre-test was 200 mm/min and 250 mm/min for the test. The BAMOnut snack bars were cut into 2.5 cm length × 1 cm height for the test. For the texture profile analysis (TPA), BAMOnut bars (4 cm length × 1 cm height) were cut with a knife and placed on the support plate and then compressed with an anvil with pre-test conditions set at 2.00 mm/s, test at 2.0 mm/s, and post-test at 2.00 mm/s.

### 2.6. Nutritional and Proximate Composition of BAMOnut Bar

#### 2.6.1. Amino Acid

The amino acid in the BAMOnut snack bar was determined using Waters Altra performance liquid chromatography (UPLC) separation with UV detection after derivatization with 6-aminoquinolyl-N-hydroxysuccinimidyl carbamate (AQC).

#### 2.6.2. Minerals

Inductively coupled plasma atomic emission spectrometry (ICP-AES) (Varian Vista-Pro axial ICP-AES instrument with SPS-5 autosampler, Varian Inc., Mulgrave, VIC, Australia) as described by [16] was followed in the determination of the mineral composition of BAMOnut Bars (oats with *Moringa*, and millet with *Moringa oleifera* leaf powder). The calibration standard was prepared using the standard procedure. About 0.5 g sample and HNO3 (5 mL) were accurately measured into the digestion vessel. The vessel was loosely capped and left to stand for 10 min at ambient temperature in a fume cupboard. The vessel was carefully closed and placed in the CEM Mars Xpress microwave. The sample was digested according to a ramp program in two stages: Stage 1—Power, expressed as maximum power (1600 watts for CEM Mars Xpress system) = 75%; ramp time, 5 min; 120 °C; hold time, 5 min; Stage 2—Power, 75%; ramp time, 5 min; 200 °C; hold time, 25 min. The samples were cooled to room temperature after digestion. The contents were quantitatively transferred to 50 mL volumetric flasks and made up to volume with deionised water. The contents of the volumetric flasks were transferred into polyethylene sample tubes for ICP-AES analysis. The mineral elements determined were sodium, magnesium, phosphorous, potassium, calcium, iron, copper and zinc.

### 2.7. Proximates

Protein determination was carried out using the Truspec Nitrogen Analyser (LECO) method, total fat according to the AOAC (2005) method was 996.06, moisture and ash according to the AOAC (2005) method were 934.01 and 923.03, respectively. The carbohydrate was determined by difference. Energy value was calculated using conversion factors of 4, 4, and 9 Kcal g^−1^ for protein, carbohydrate, and lipids, respectively.

### 2.8. Sensory

A benchtop sensory evaluation was conducted using 10 panellists consisting of students and staff of the Cape Peninsula University of Technology, who were served 3 samples of BAMOnut Bar. The bars were rectangular and ranged from 58.99 to 48.04 g in size. The BAMOnut bars were produced 4 h before the sensory evaluation. The samples were presented in a zip-locked bag with three-digit codes. A cup of water was provided to reset the palate in-between the tastings. A score sheet was given to each panellist that consisted of three-digit coded samples with a 5-point hedonic scale ranging from 1 = dislike extremely to 5 = like extremely [17]. They were instructed to rate each sample individually on its merit on a five-point hedonic scale rating for appearance, colour, taste, aroma, texture, and overall acceptability. The ten (10) panellists comprised 30% males and 70% females, 20% were staff, and 80% were students. The age categories of the panellists were as follows; less than 20 years (20%), 20–29 years (40%), 30–39 years (30%), and over 40 years (10%).

### 2.9. Statistical Analysis

All experiments were conducted in triplicate. To determine mean differences between treatments, obtained data were subjected to multivariate analysis of variance (ANOVA). Where differences existed, separation of means was carried out using Duncan’s multiple range test. The principal component analysis (PCA) on the correlation matrix with eigenvalues over 1 was used to establish the components that accounted for the variation in the physicochemical, textural and fatty acid profiles of the bars (IBM SPSS, version 25).

## 3. Results and Discussion

### 3.1. Physical Characteristics of BAMOnut Bar

#### 3.1.1. Physical Appearance

**Figure 2:**
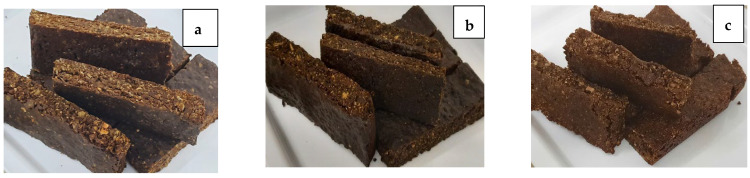
Physical appearance of BAMOnut snack bars—(**a**): BAMOnut enriched with oats and 3% BAMOLP (BAMOnut-OB3), (**b**): BAMOnut enriched with millet and 2% BAMOLP (BAMOnut-MB2), and (**c**): BAMOnut enriched with oats, millet, and 5% BAMOLP (BAMOnut-OMB5).

Images of BAMOnut snack bars are presented in Figure 2. BAMOnut bars enriched with oats and 3% BAMOLP (BAMOnut-OB3), shown in Figure 2a, appeared more attractive and firmer than BAMOnut bars enriched with millet and 2% BAMOLP (BAMOnut-MB2) and BAMOnut bars enriched with oats, millet, and 5% BAMOLP (BAMOnut-OMB5). This difference could be due to the absence of water in BAMOnut-OB3, whereas cooked and wet millet was incorporated in the production of BAMOnut-MB2 and BAMOnut-OMB5. The wetness of the cooked millet may have contributed to the reduced firmness in both BAMOnut-MB2 and BAMOnut-OMB5.

#### 3.1.2. Colour Attributes

The colour of BAMOnut snack bars is shown in Table 4. The average lightness for BAMOnut-OB3 (BAMOnut snack bar enriched with oats, and 3% BAMOLP), BAMOnut-MB2 (BAMOnut snack bar enriched with millet, and 2% BAMOLP), and BAMOnut-OMB5 (BAMOnut snack bar enriched with oats, millet, and 5% BAMOLP) were 25.82, 19.16, and 21.60 respectively. The redness of BAMOnut-OB3, BAMOnut-MB2, and BAMOnut-OMB5 was 7.23, 3.74, and 4.66, respectively. The yellowness of BAMOnut-OB3, BAMOnut-MB2, and BAMOnut-OMB5 was 19.53, 11.08, and 13.08 respectively, and the chroma were 20.83, 11.70, and 14.59, respectively, while the hue angles were 69.67°, 71.24°, and 71.34° respectively. All the bars differed significantly (*p* < 0.05) in lightness, redness, yellowness, and chroma. The hue angle of BAMOnut snack bars did not differ significantly.

Chroma describes the intensity of colour perceived by humans [12]; the BAMOnut snack bars were not vivid, their chroma ranged from 11.70 to 20.83 which is far from the saturation point of 120. Hue is how the colour of an object is perceived.

The hue angle of the bars indicated that BAMOnut bars are dominated by a yellowish colour as they are close to a hue-angle of 90^o^, which represents pure yellowness (chroma 11.7–20.83, hue; 69.67–71.34). BAMOnut-OB3 bar produced with oat and 3% BAMOLP had a higher L*. The higher L* is an indication of greater lightness than the other two bars. Nevertheless, all the three bars can be described as dark in colour, as they are closer to zero which indicates black on the colour scale (19.2 to 25.8). Hence, the BAMOnut bar can be described as dark unsaturated yellowish-red.

The external appearance of the BAMOnut snack bars showed a darker colour (Figure 2) which could be a result of Maillard and caramelization reactions between protein, sugar, and honey in the formulation during baking [18]. Colour is a key attribute that influences consumer acceptability of a food product at the time of purchase [12].

The colour differences (ΔE) between BAMOnut-OB3 and BAMOnut-MB2, BAMOnut-MB2 and BAMOnut-OMB5, and BAMOnut-OB3 and BAMOnut-OMB5 were determined to be 27.89, 4.10, and 7.69, respectively. The colour differences were perceivable since a colour difference > 1 is defined as a just noticeable difference, where the observer can notice the difference. The sensory evaluation confirmed that the consumers perceived differences in colour of the BAMOnut snack bars, and the snack bars were acceptable. Other researchers [19] also produced acceptable ready-to-eat snacks with alkali pre-treated 20% Moringa leaf flour. BAMOnut-snack bar produced with oats and 3%, BAMOLP (BAMOnut-OB3) may be preferred because it is the lightest of the three variations.

#### 3.1.3. Microstructure of BAMOnut Snack Bars

**Figure 3:**
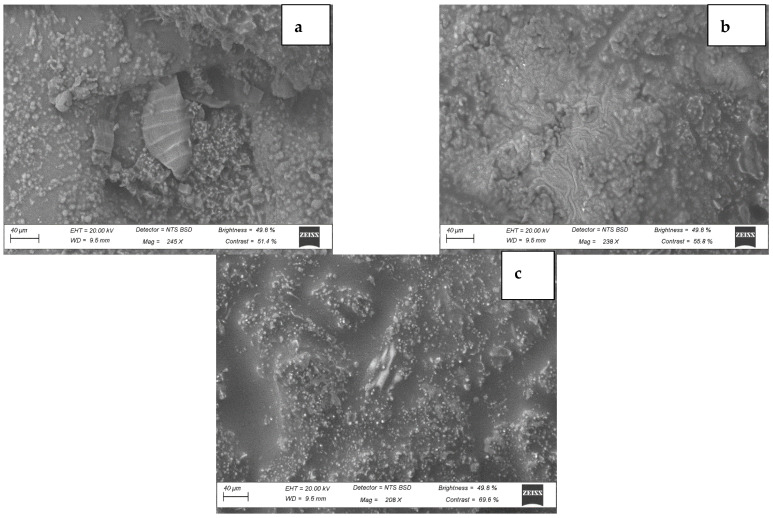
Scanning Electron Micrograph of Bambara groundnut-*Moringa oleifera* snack bars (BAMOnut), showing (**a**) morphology of BAMOnut enriched with oats and 3% BAMOLP, (**b**) BAMOnut enriched with millet and 2% BAMOLP, (**c**) BAMOnut enriched with oats, millet and 5% BAM BAMOLP, (magnification ×245 for (**a**), (**b**) ×238, (**c**) ×206). All the micrographs are on a scale of 40 µm.

**Figure 4:**
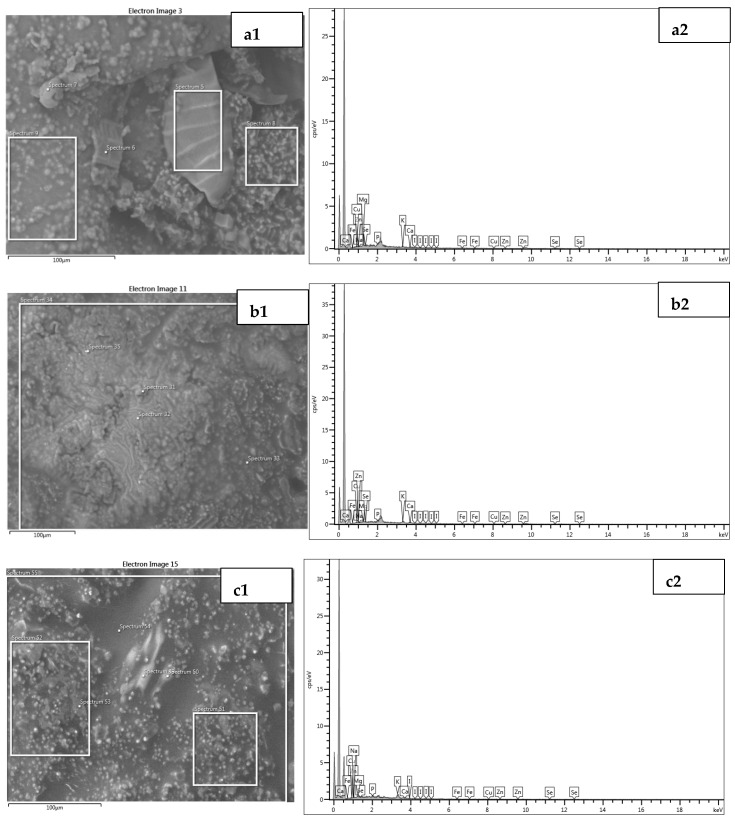
EDX characterization spectrum obtained for BAMOnut snack bars: (**a**) BAMOnut enriched with oats and 3% BAMOLP, (**b**) BAMOnut enriched with millet and 2% BAMOLP, (**c**) BAMOnut enriched with oats, millet and 5% BAMOLP. All the micrographs are on a scale of 100 µm. Visible peaks confirm the presence of sodium, magnesium, phosphorous, potassium, calcium, iron, nickel, copper, zinc, selenium, iodine.

Scanning electron micrographs (SEM) of BAMOnut snack bars are shown in Figure 3, the particles are in micrometre scales (40 µm) and agglomerated. The BAMOnut snack bars had particles with irregular shapes and different sizes. BAMOnut-OB3 had larger particles compared with BAMOnut-MB2 and BAMOnut-OMB5. The quantitative analysis using energy dispersion X-ray diffraction (EDX) shown in Figure 4 indicated that 11 elements were present in the BAMOnut snack bars. The EDX spectrum showed the presence of sodium (0.7–9.7%), magnesium (5.4–15.7%), phosphorous (17.3–25.1%), potassium (24.7–30.4%), calcium (5.2–22.8%), iron (1.4–1.8%), nickel (1.2–3.2%), copper (4.1–16.3%), zinc (0.3–5.3%), selenium (0.7–3.1%), and iodine (2.7–9.4%). The elements present are expressed in weight percentage and in proportion to the total elements of the area analysed.

**Figure 5:**
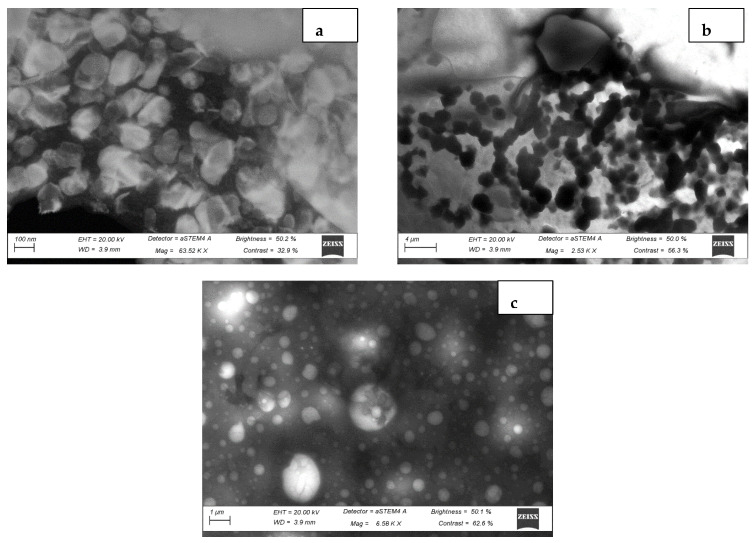
Scanning Transmission Electron Micrograph of Bambara groundnut-*Moringa oleifera* bars (BAMOnut). (**a**) Morphology of BAMOnut enriched with oats and 3% BAMOLP on 100 nm scale, (**b**) BAMOnut enriched with millet and 2% BAMOLP on 4 µm scale, (**c**) BAMOnut enriched with oats, millet and 5% BAMOLP on 1 µm scale (magnification 63.52 KX for (**a**), (**b**) 2.53 KX, (**c**) 6.58 KX).

Scanning transmission electron microscopy (STEM) further showed the variation of the internal structure of the snack bars (Figure 5). BAMOnut-MB2 and BAMOnut-OMB5 showed more uniform microstructure compared to OB3, this is possibly due to the fact that both MB2 and OMB5 contain cooked millet while OB3 contains roasted oats. The availability of extra water molecules for absorption by the ingredients of MB2 and OMB5 may have enhanced their uniformity. It is worth mentioning that individual components of the snack bars such as proteins, starch, and crystals are clearly distinguishable as there was no hydrocolloid in the formulation which could promote even distribution of the components. According to [20], the addition of hydrocolloids to cereal bars leads to more homogeneous microstructure.

#### 3.1.4. Water Activity Characteristics of Bambara Groundnut-*Moringa oleifera* Snack Bar

The water activity of the three BAMOnut bars (OB3, MB2, OMB5) is shown in Table 5. The water activities of the BAMOnut snack bars were 0.34, 0.57, and 0.58, respectively, which is comparable to the 0.6 maximum standard requirement for RUTF. The BAMOnut-OB3 has the least water activity, significantly (*p* < 0.05) different from BAMOnut-MB2 and BAMOnut-OMB5. However, there was no significant difference between the water activity of BAMOnut-MB2 and BAMOnut-OMB5, and their water activity compares with that of date and apricot bars (0.534–0.546) [21]. Water activity is an indicator of available water in food, and is a useful tool for prediction of food quality and storage stability. The water activity levels of BAMOnut-MB2 and BAMOnut-OMB5 were higher due to the addition of cooked millet to both formulations, nevertheless, they were still within 0.6 max requirement for RUTF. The low water activity of the BAMOnut bars suggests that there will be retarded growth of microorganisms in the products, which implies good storage stability and long shelf life. BAMOnut snack bar enriched with oats and 3% BAMOLP (BAMOnut-OB3) was the best in terms of water activity and was also considered best in colour.

#### 3.1.5. Textural Properties Profile of BAMOnut Bar

Texture is an important parameter associated with food products. It is the sensory manifestation of the structure of a food and reveals the mode by which the structure responds to an applied force. Textural parameters tested were cut (shear) test, puncture test, springiness, hardness and gumminess (Table 6). BAMOnut-OB3 was significantly (*p* < 0.05) higher in cutting energy, puncture energy and hardness than BAMOnut-MB2 and BAMOnut-OMB5, but there was no significant difference in hardness of BAMOnut-MB2 and BAMOnut-OMB5. BAMOnut-OB3 was not springy, and there was no significant difference between BAMOnut-MB2 and BAMOnut-OMB5 in terms of springiness and gumminess. The higher hardness of BAMOnut-OB3 can be linked to its lower moisture content, compared with BAMOnut-MB2 and BAMOnut-OMB5 in which cooked millet was added to the formulation.

The higher hardness of OB3 explains its higher cutting and higher puncture energy, which can further be linked to the addition of roasted oats to the formulation, compared with MB2 and OMB5 in which cooked wet millet was added. The bulkiness of dry ingredients enhances proper absorption of wet ingredients, which results in low moisture content and a firmer product. It is therefore not surprising that MB2 and OMB5 were gummier, required less energy for cutting and had less puncture strength, due to insufficient dry ingredients in the formulation.

### 3.2. Proximate and Amino Acid Composition of Bambara Groundnut-Moringa oleifera Snack Bar

#### 3.2.1. Amino Acid Composition of Bambara Groundnut-*Moringa oleifera* Snack Bar

The amino acid composition of BAMOnut-OB3 (BAMOnut bar enriched with oats and 3% BAMOLP), BAMOnut-MB2 (BAMOnut bar enriched with millet and 2% BAMOLP), and BAMOnut-OMB5 (BAMOnut bar enriched with oats, millet, and 5% BAMOLP), are presented in Table 7. The total amino acids in BAMOnut-OB3, BAMOnut-MB2, and BAMOnut-OMB5 were 15.65, 14.82, and 11.70 g/100 g respectively. The essential amino acids of the aforementioned BAMOnut bars were 38.47, 39.34, and 37.01% of the total amino acids, respectively. BAMOnut-OB3 and BAMOnut-MB2 snack bars were significantly (*p* < 0.05) higher in threonine, phenylalanine, histidine, valine, isoleucine, and leucine in comparison to BAMOnut-OMB5 (Table 7). Tryptophan was lost during acid hydrolysis of the protein, hence it was absent in all the samples.

The non-essential amino acids of BAMOnut-OB3, BAMOnut-MB2, and BAMOnut-OMB5 snack bars were 61.53, 60.66, and 62.99% of the total amino acids, respectively. Major non-essential amino acids observed were glutamine, arginine, and asparagine. Arginine was significantly higher in BAMOnut-OB3 and BAMOnut-MB2 than in BAMOnut-OMB5; arginine has been reported to prevent heart disease [22]. BAMOnut-OB3 and BAMOnut-OMB5 had significantly higher asparagine and alanine than BAMOnut-MB2. Glutamine was higher in BAMOnut-MB2 and BAMOnut-OMB5. Cysteine was not detected in the BAMOnut snack bar samples.

Amino acids are organic compounds, which are precursors of proteins; therefore, they influence the quantity and quality of protein [8,23,24]. Amino acids are categorised as essential and non-essential and vary according to animal species and their production system. They are indispensable in the production of enzymes, immunoglobins, hormones, and the growth and repair of body tissues, and they form the structure of red blood cells [8]. Furthermore, they play an important role in the formation of glucose, acting as a buffer when other precursors are in short supply. Amino acids are essential for the performance of specific functions in the body [8]. BAMOnut-OB3 and BAMOnut-MB2 showed a better amino acid profile compared with BAMOnut-OMB5 (Table 7). Although there was no significant difference between BAMOnut-OB3 and BAMOnut-MB2 in some amino acids, BAMOnut-OB3 had a higher content of phenylalanine, histidine, serine, glycine, glutamine, proline, and tyrosine. The BAMOnut snack bar enriched with oats and 3% BAMOLP (BAMOnut-OB3) can therefore be considered the best in terms of amino acid profile, the same conclusion as was reached for colour and water activity.

#### 3.2.2. Mineral Composition

The mineral compositions of BAMOnut snack bar enriched with oats and 3% BAMOLP (OB3), and BAMOnut snack bar enriched with millet and 2% BAMOLP (MB2) are presented in Table 8. Two varieties of the bars were selected for mineral analysis, to reduce costs. The two varieties analysed were chosen for their better amino acid profiles and chemical composition compared to the third variety (Table 7 and Table 9). The mineral compositions in the BAMOnut snack bar are comparable to the requirements for ready-to-use therapeutic food (RUTF), except for magnesium, potassium and zinc. In comparison to RUTF, magnesium levels in BAMOnut snack bar are higher while potassium and zinc are lower (Table 8). This implies that utilisation of cereals and legumes in the formulation can successfully replace the mineral and vitamin pack used in RUTF.

#### 3.2.3. Chemical Composition of BAMOnut Snack Bar

The chemical composition of BAMOnut-OB3 (BAMOnut Bar enriched with oats and 3% BAMOLP), BAMOnut-MB2 (BAMOnut Bar enriched with millet and 2% BAMOLP), and BAMOnut-OMB5 (BAMOnut Bar enriched with oats, millet, and 5% BAMOLP), are displayed in Table 9. The moisture contents of BAMOnut-OB3, BAMOnut-MB2, and BAMOnut-OMB5 were 4.9, 7.9, and 5.8% respectively. The moisture contents of the BAMOnut snack bars differed significantly (*p* < 0.05) from each other. The moisture content was higher than 2.5% required for RUTF, which may be due to the high moisture contents of some ingredients used in the formulation (8.0, 5.0, 4.7, 5.2, and 8.0% for oats, millet, BAMOLP, melon seed, and MOLP respectively). However the moisture content is comparable to the moisture content of snack bars reported by some researchers ([21,25]), and is lower than the granola experimental bar produced by [26]. Seeing that the moisture content of BAMonut is comparable to previous work on cereal bars, and is even lower than some, it can be concluded that BAMOnut snack bars will be shelf-stable because lower moisture content will enhance product stability. Formulations with millet had higher moisture because the millet was cooked and was used wet. BAMOnut-OB3 had the least moisture content, and hence long shelf-life and low susceptibility to microbial spoilage.

The protein content of the BAMOnut snack bars was 14.1, 14.8, and 11.4% for BAMOnut-OB3, BAMOnut-MB2, and BAMOnut-OMB5, respectively; values that were lower than the optimisation goal (24.0, 27.0, and 22.6%). There was no significant difference between the protein content of BAMOnut bars enriched with oats and 3% BAMOLP (BAMOnut-OB3), and those enriched with millet and 2% BAMOLP (BAMOnut-MB2), but both were significantly (*p* < 0.05) different from BAMOnut-OMB5 in protein content. The highest protein content was observed at 2% (low concentration) of BAMOLP, in combination with a high concentration of both egusi and MOLP. The protein content decreased with increased concentration of BAMOLP; this could be due to synergistic effects of the other ingredients, especially egusi and MOLP which decreased as BAMOLP increased. However, there was no significant difference in protein content when BAMOLP was added at low and medium concentrations. Nevertheless, the protein content increased with an increase in the concentration of *Moringa oleifera* leaf powder (MOLP). It was reported that the protein content of ‘ogi’ increased when MOLP was added, with similar results for supplementation of wheat flour with MOLP in bread [27]. However, the addition of the Bambara groundnut-*Moringa oleifera* leaf protein complex (BAMOLP) resulted in an insignificant increase in protein content because the formulation with the highest BAMOLP had the least protein content, but with a better amino acid profile. This may be because BAMOLP was added in small quantities, insufficient to produce a significant effect. As per RUTF requirements, the protein-calorie contribution is expected to be 10–12% of the total energy. All three variations of the BAMOnut snack bar met this standard, 12.0, 13.8, 10.4% of total energy for BAMOnut-OB3, BAMOnut-MB2, and BAMOnut-OMB5 respectively.

The fat content of the BAMOnut snack bars was 19.3, 14.7, and 13.2% for BAMOnut-OB3, BAMOnut-MB2, and BAMOnut-OMB5 respectively. There was no significant (*p* > 0.05) difference between the fat content of BAMOnut snack bar enriched with millet and 2% BAMOLP (BAMOnut-MB2), and those enriched with oats, millet, and 5% BAMOLP (BAMOnut-OMB5), but both were significantly (*p* < 0.05) different from BAMOnut-OB3 in fat content. The difference in the fat content could be a result of cooked millet, which is low in fat (1.7% fat content), being added to the formulations of BAMOnut-MB2 and BAMOnut-OMB5, while oats (6.9% fat content) were added in the formulation of BAMOnut-OB3. The fat content of RUTF is expected to be 45–60% of the total energy; 37.0, 30.8, and 27.2% total energy was obtained for BAMOnut-OB3, BAMOnut-MB2, and BAMOnut-OMB5, respectively. A reduction in the concentration of honey from 45% to 30%, and an increase in canola oil from 15% to 30% is likely to increase the fat content of the BAMOnut snack bar. The reasoning is because honey mainly contributed to carbohydrate content, which was higher than that required for RUTF in all three variations.

The ash content of BAMOnut bars differed significantly (*p* < 0.05) from each other. BAMOnut-MB2 (3.04%) had the highest content of ash, followed by BAMOnut-OB3 (2.06), and BAMOnut-OMB5 (1.57). The ash content was observed to increase with the elevated concentration of MOLP in the formulations (Table 9). Several studies have revealed that *Moringa oleifera* leaf powder is high in ash contents, ranging from 4.6–10.9% [27,28,29,30,31,32,33]. Ash content is an indication of the mineral matter present in the food substance [31].

There was no significant (*p* > 0.05) difference between the carbohydrate composition of BAMOnut-OB3 and BAMOnut-MB2, but both differed (*p* < 0.05) significantly from BAMOnut-OMB5. The difference is probably due to the combination of oats and millet used in BAMOnut-OMB5. The carbohydrate content of BAMOnut snack bars was higher than the recommended value for RUTF (41–58%) but is comparable to previous research work on cereal bars.

The energy values of the BAMOnut snack bars were 468.6, 429.4, and 436.4 Kcal for BAMOnut-OB3, BAMOnut-MB2, and BAMOnut-OMB5, respectively. The energy content of BAMOnut-OB3 was significantly (*p* < 0.05) higher than the content of BAMOnut-MB2 and BAMOnut-OMB5. However, there was no significant (*p* > 0.05) difference between the energy content of BAMOnut-MB2 and BAMOnut-OMB5. The difference in energy is due to the difference in fat content of the BAMOnut snack bars; fat provides a high coefficient of nutritional values (9 Kcal) compared to protein or carbohydrate which provide a lower coefficient of nutritional values (4 Kcal). The energy content of BAMOnut snack bars was lower than the recommended value (520–550) Kcal for RUTF, but comparable to the energy value of cereal bars in previous studies [26]. Therefore, BAMOnut snack bars could be classified as a high-calorie snack bar.

### 3.3. Sensory

**Figure 6:**
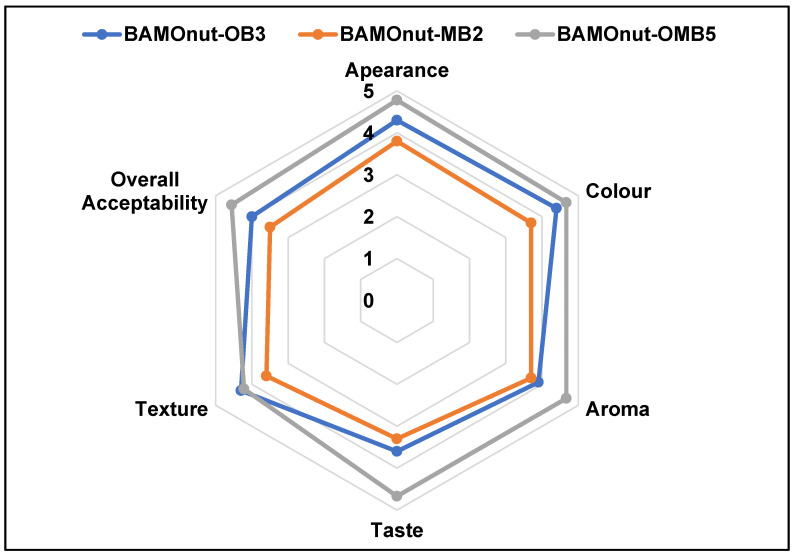
Spider plot showing sensory scores for Bambara groundnut-*Moringa oleifera* snack bars (BAMOnut).

Sensory scores for sensorial parameters and overall acceptability of BAMOnut snack bars are presented in Figure 6. The panellists’ mean ratings for appearance, colour, aroma, taste, texture and overall acceptability of BAMOnut-OB3, BAMOnut-MB2 and BAMOnut-OMB5 were illustrated in Figure 6.

Two of the BAMOnut snack bars (BAMOnut-OB3 and BAMOnut-OMB5) were not significantly different for appearance, colour, and aroma, but both were significantly (*p* < 0.05) different to BAMOnut-MB2. The appearances of BAMOnut-OB3 and BAMOnut-OMB5 (4.30, 4.78) were judged significantly (*p* < 0.05) higher compared to that of BAMOnut-MB2 (3.80). The colours of BAMOnut-OB3 and BAMOnut-OMB5 (4.40, 4.67) scored significantly (*p* < 0.05) higher in comparison to BAMOnut-MB2 (3.70). The panellists described the colour of BAMOnut-MB2 as very dark. The aroma of BAMOnut-OB3 and BAMOnut-OMB5 (3.90), (4.67) were rated significantly (*p* < 0.05) higher compared to that of BAMOnut-MB2 (3.70). The bar (BAMOnut-MB2) that included millet and 2% BAMOLP had lower ratings for appearance, colour, and aroma due to the high concentration (80 g) of MOLP used in the formulation. A similar observation was reported by [27] for MOLP supplementation in wheat bread.

BAMOnut-OB3 and BAMOnut-MB2 were not significantly (*p* > 0.05) different in terms of taste and overall acceptability. The tastes of BAMOnut-OB3 and BAMOnut-MB2 were rated significantly (*p* < 0.05) lower (3.60, 3.30) than that of BAMOnut-OMB5 (4.67). Some of the panellists described the taste of BAMOnut-MB2 as slightly bitter while some described it as a mild burnt taste, due to the high concentration of MOLP in the formulation. Statistically, there was no significant difference in the texture of all three variants of the BAMOnut snack bar. The overall acceptability of BAMOnut-OB3 and BAMOnut-MB2 was significantly (*p* < 0.05) lower than BAMOnut-OMB5 (4.56), a difference that can be attributed to the high concentration of MOLP in the formulations. The panellists described BAMOnut-OB3 as too sweet, nice, and crunchy, and a little bit hard, and BAMOnut-OMB5 as too sweet, soft, and crunchy and delicious, while BAMOnut-MB2 was described as having a very dark colour, a mild burnt taste, a slightly bitter taste, bitter, strong aroma, soft and crumbly.

### 3.4. The Principal Component of the Bambara Groundnut-Moringa oleifera Snack Bar Sensory

**Figure 7:**
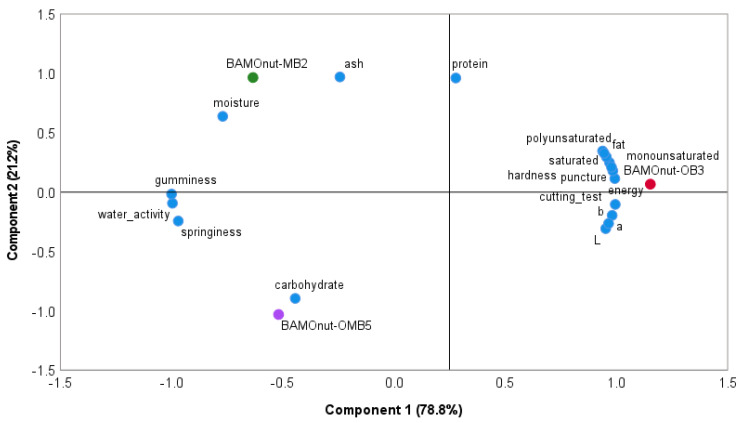
Principal components of Bambara groundnut-*Moringa oleifera* snack bars.

Principal component analysis (PCA) on the physical and proximate properties of Bambara groundnut-*Moringa oleifera* snack bar data is presented in Figure 7. The first and second principal components represented 78.8% and 21.2% of the observed variation, respectively. PC1 separated the BAMOnut-bars, with the BAMOnut-MB2 and BAMOnut-OMB5 on the top right and bottom right respectively, and the BAMOnut-OB3 nearer the middle on the right. BAMOnut-OB3 was characterised by higher fat (mono and poly fat), cutting, energy, and lightness, BAMOnut-MB2 by ash content and moisture, and BAMOnut-OMB5 by carbohydrate. Yellowness and protein content can be attributed to both OB3 and MB2. The difference in fat content of BAMOnut-OB3 compared to BAMOnut-MB2 and BAMOnut-OMB5 may be due to the high content of fat in oats (8%) compared to 4.8% of fat in millet. High-fat content leads to high energy. The lightness of BAMOnut-OB3 could be a result of a decreased concentration of MOLP in the formulation compared to BAMOnut-MB2, and variation of ingredients compared to OMB5. The ash content, which is an indication of mineral presence, was higher in BAMOnut-MB2 due to the higher concentration of MOLP in that formulation compared to BAMOnut-OB3 and BAMOnut-OMB5, and its moisture content was higher due to the cooked millet in the formulation. BAMOnut-OB3 and BAMOnut-MB2 can be considered better formulations than BAMOnut-OMB5. The moisture, energy, and fat content of BAMOnut-OB3 were closer to the required standards for RUTF compared to those of BAMOnut-MB2, while the colour of BAMOnut-OB3 was better, which is an important attribute for consumer acceptability of a food product. Although BAMOnut-MB2 was high in ash content, its moisture content was high, and it will be safer to choose a formulation with lower moisture content to guarantee good shelf life and a high-quality product. BAMOnut-OB3 could therefore be considered as the best formulation for the BAMOnut-snack bar, which was also confirmed by the water activity described in Section 3.1.4 and amino acid content in Section 3.2.1.

## 4. Conclusions

This study has confirmed that legumes and cereals can be used to produce ready-to-use therapeutic food (RUTF) that will meet WHO requirements. BGN and *Moringa oleifera* leaf powder with the addition of egusi, oats, and millet were successfully used to produce snack bars with acceptable sensory scores and nutritional values. The BAMOnut snack bar produced with oats can be considered as the best formulation. The protein content of the BAMOnut snack bars ranged from 11.4–14.8%. The protein content of BAMOnut-snack bars (OB3 and MB2) is comparable to the 13–16% required for RUTF, which implies that RUTF can be produced without the addition of full milk powder as a standard RUTF ingredient. The low water activity of the bars indicates low risk of microbial growth. The BAMOnut snacks may serve as an alternative RUTF for severely acutely malnourished children and also as nutritious snacks for adults [6].

## Figures and Tables

**Table 1:** Proximate composition of ingredients.

	Fat	Protein	Water	Others	Reference
BAMOLP	8.07	63.51	2.42	26.0	[1]
Egusi	49.0	28.0	5.10	17.9	[7]
MOLP	6.0	33.3	5.6	55.1	[8]
Oats	6.52	13.15	10.84	69.49	[9]
Millets	3.33	10.00	4.00	82.67	[10]

BAMOLP: Bambara groundnut-*Moringa oleifera* protein complex, MOLP: *Moringa oleifera* leaf powder.

**Table 2:** Optimisation goal for Bambara groundnut and *Moringa oleifera* snack bar.

Chemical Composition (%)	OB3	MB2	OMB5
Fat	26	26	24.9
Protein	24	27	22.6
Water	7.29	5	5.9

BAMOLP: Bambara groundnut-*Moringa oleifera* protein complex, OB3: BAMOnut snack bar enriched with oats and 3% BAMOLP, MB2: BAMOnut snack bar enriched with millet and 2% BAMOLP, OMB5: BAMOnut snack bar enriched with oats, millet, and 5% BAMOLP.

**Table 3:** Formulation of three varieties of BAMOnut-Bar.

Ingredients (%)	OB3	MB2	OMB5
BAMOLP	3.0	2.0	5.0
Egusi	46.0	48.0	45.0
MOLP	13.0	32.0	5.0
Oats	38.0.0	-	20.0
Millet	-	19.0	25.0

BAMOnut: Bambara groundnut-*Moringa oleifera* snack bar, OB3: BAMOnut snack bar enriched with oats and 3% BAMOLP, MB2: BAMOnut snack bar enriched with millet and 2% BAMOLP, OMB5: BAMOnut snack bar enriched with oats, millet, and 5% BAMOLP. BAMOLP: Bambara groundnut-*Moringa oleifera* protein complex, MOLP: *Moringa oleifera* leaf powder.

**Table 4:** Colour attributes of Bambara groundnut-*Moringa oleifera* snack bar.

BAMOnut Bar	Colour Parameters				
	L*	a*	b*	C*	h°
OB3	25.82 ± 0.39 ^a^	7.23 ± 0.15 ^a^	19.53 ± 1.15 ^a^	20.83 ± 1.13 ^a^	69.67 ± 0.70 ^a^
MB2	19.16 ± 0.07 ^c^	3.74 ± 0.20 ^c^	11.08 ± 1.32 ^c^	11.70 ± 3.56 ^c^	71.24 ± 1.19 ^a^
OMB5	21.60 ± 0.55 ^b^	4.66 ± 1.95 ^b^	13.83 ±1.09 ^b^	14.59 ± 1.03 ^b^	71.34 ± 1.25 ^a^

Mean values ± standard deviation of triplicate determination. Mean values in the same column followed by different letters are significantly (*p* < 0.05) different. L*: Lightness; a*: Redness; b*: Yellowness; C*: Chroma; h°: Hue angle; BAMOnut: Bambara groundnut-*Moringa oleifera* snack bar; OB3: BAMOnut snack bar enriched with oats and 3% BAMOLP; MB2: BAMOnut snack bar enriched with millet and 2% BAMOLP; OMB5: BAMOnut snack bar enriched with oats, millet, and 5% BAMOLP.

**Table 5:** Water activity of Bambara groundnut-*Moringa oleifera* snack bars.

BAMOnut Variety	Water Activity
OB3	0.34 ± 0.01 ^a^
MB2	0.57 ± 0.01 ^b^
MMB5	0.58 ± 0.00 ^b^

Mean values ± standard deviation of triplicate determination. Mean values in the same column followed by different letters are significantly (*p* < 0.05) different, BAMOnut: Bambara groundnut-*Moringa oleifera* snack bar, OB3: BAMOnut snack bar enriched with oats and 3% BAMOLP, MB2: BAMOnut snack bar enriched with millet and 2% BAMOLP, OMB5: BAMOnut snack bar enriched with oats, millet, and 5% BAMOLP.

**Table 6:** Textural profile of Bambara groundnut-*Moringa oleifera* snack bars.

BAMOnut Bars	Cut (Shear) N	Punture (N)	Springiness (mm)	Hardness (N)	Gumminess (N)
OB3	127.86 ± 41.90 ^a^	45.56 ± 9.41 ^a^	0.00 ± 0.00 ^a^	500.56 ± 0.47 ^a^	0.00 ± 0.00 ^a^
MB2	25 10 ± 1.98 ^b^	8.35 ± 1.88 ^b^	2.56 ± 0.24 ^b^	341.05 ± 21.14 ^b^	673.23 ± 26.72 ^b^
OMB5	18.13 ± 4.44 ^c^	2.68 ± 0.24 ^c^	3.19 ± 0.86 ^b^	300.24 ± 37.08 ^b^	642.38 ± 24.68 ^b^

Mean values ± standard deviation of triplicate determination. Mean values in the same column followed by different letters are significantly (*p* < 0.05) different, BAMOnut: Bambara groundnut-*Moringa oleifera* snack bar, OB3: BAMOnut snack bar enriched with oats and 3% BAMOLP, MB2: BAMOnut snack bar enriched with millet and 2% BAMOLP, OMB5: BAMOnut snack bar enriched with oats, millet, and 5% BAMOLP.

**Table 7:** Amino acid composition of BAMOnut.

Amino Acid (g/100 g)	BAMOnut Bar		
Essential	OB3	MB2	OMB5
Threonine	0.87 ± 0.02 ^a^	0.82 ± 0.02 ^a^	0.53 ± 0.01 ^b^
Methionine	0.26 ± 0.00	0.29 ± 0.00	0.14± 0.00
Phenyalanine	1.60 ± 0.02 ^a^	1.43 ± 0.02 ^b^	1.05 ± 0.01 ^c^
Histidine	0.57 ± 0.01 ^a^	0.50 ± 0.01 ^b^	0.37 ± 0.00 ^c^
Lysine	0.12 ± 0.00	0.22 ± 0.00	0.26 ± 0.00
Valine	0.77 ± 0.01 ^a^	0.75 ± 0.00 ^a^	0.57 ± 0.01 ^b^
Isoleusine	0.65 ± 0.01 ^a^	0.66 ± 0.01 ^a^	0.50 ± 0.01 ^b^
Leucine	1.18 ± 0.03 ^a^	1.16 ± 0.02 ^a^	0.91 ± 0.01 ^b^
Non-Essential			
Serine	1.08 ± 0.01 ^a^	0.96 ± 0.01 ^b^	0.71 ± 0.01 ^c^
Arginine	1.85 ± 0.03 ^a^	1.86 ± 0.04 ^a^	1.41 ± 0.01 ^b^
Glycine	1.03 ± 0.01 ^a^	0.97 ± 0.01 ^b^	0.64 ± 0.01 ^c^
Asparagine	1.13 ± 0.01 ^a^	1.25 ± 0.00 ^b^	1.13 ± 0.02 ^a^
Glutamine	2.40 ± 0.05 ^a^	2.12 ± 0.02 ^b^	2.09 ± 0.03 ^b^
Alanine	0.55 ± 0.01 ^a^	0.61 ± 0.01 ^b^	0.52 ± 0.01 ^a^
Proline	0.59 ± 0.01 ^a^	0.54 ± 0.01 ^b^	0.46 ± 0.01 ^c^
Tyrosine	1.00 ± 0.01 ^a^	0.68 ± 0.01 ^b^	0.41 ± 0.00 ^c^
TAA	15.65	14.82	11.70
TEAA	6.02	5.83	4.33
TNAA	9.63	8.99	7.37
%TEAA/TAA	38.47	39.34	37.01
%TNAA/TAA	61.53	60.66	62.99

Mean values ± standard deviation of triplicate determination. Mean values in the same row followed by different letters are significantly (*p* < 0.05) different, BAMOnut: Bambara groundnut-*Moringa oleifera* snack bar, OB3: BAMOnut snack bar enriched with oats and 3% BAMOLP, MB2: BAMOnut snack bar enriched with millet and 2% BAMOLP, OMB5: BAMOnut snack bar enriched with oats, millet, and 5% BAMOLP. TAA: Total amino acid, TEAA: Total essential amino acid, TNAA: Total non-essential amino acid.

**Table 8:** Mineral composition of BAMOnut.

Mineral (mg/100 g)	BAMOnut Bar		
	OB3	MB2	RUTF (per 100 g)
Sodium	11.10 ± 0.14 ^a^	25.00 ± 1.13 ^b^	< 290 mg
Magnesium	168.50 ± 2.12 ^a^	220 ± 2.83 ^b^	80–140 mg
Phosphorous	267.50 ± 7.78 ^b^	238.50 ± 2.12 ^a^	300–600 mg
Potassium	360.50 ± 6.36 ^a^	467.50 ± 4.95 ^b^	1100–1400 mg
Calcium	243.50 ± 3.54 ^a^	536.50 ± 7.78 ^b^	300–600 mg
Iron	7.68 ± 0.25 ^a^	11.75 ± 1.48 ^a^	10–14 mg
Copper	0.78 ± 0.02 ^a^	0.80 ± 0.00 ^a^	1.4–1.8 mg
Zinc	1.61 ± 0.08 ^a^	1.37 ± 0.15 ^a^	11–14 mg

Mean values ± standard deviation of triplicate determination. Mean values in the same row followed by different letters are significantly (*p* < 0.05) different, BAMOnut: Bambara groundnut-*Moringa oleifera* snack bar, OB3: BAMOnut snack bar enriched with oats and 3% BAMOLP, MB2: BAMOnut snack bar enriched with millet and 2% BAMOLP.

**Table 9:** Chemical composition (g/100 g) of BAMOnut bar (OM, MM, and OMM).

Proximate (%)	OB3	MB2	OMB5	RUTF Requirement
Moisture	4.9 ± 0.2 ^a^	7.9 ± 0.6 ^c^	5.8 ± 0.1 ^b^	2.5
Protein	14.1 ± 0.9 ^a^	14.8 ± 0.9 ^a^	11.4 ± 0.5 ^b^	13–16% by weight
	12.04	13.78	10.45	10–12% total energy
Ash	2.06 ± 0.04 ^a^	3.04 ± 0.03 ^b^	1.57 ± 0.01 ^c^	-
Fat	19.3 ± 1.2 ^a^	14.7 ± 0.9 ^b^	13.2 ± 0.2 ^b^	26–36% by weight
	37.16	30.79	27.22	45–60% total energy
Saturated	4.9 ± 0.4 ^a^	3.7 ± 0.1 ^b^	3.2 ± 0.1 ^c^	-
Monounsaturated	6.4 ± 0.7 ^a^	4.9 ± 0.1 ^b^	4.6 ± 0.1 ^b^	-
Polyunsaturated	7.9 ± 0.9 ^a^	6.1 ± 0.1 ^b^	5.4 ± 0.1 ^b^	-
Carbohydrates	59.7 ± 1.1 ^a^	59.6 ± 0.4 ^a^	68.1 ± 0.3 ^b^	41–58
Energy (Kcal)	468.6 ± 10.7 ^a^	429.7 ± 3.2 ^b^	436.4 ± 0.84 ^b^	520–550

Mean values ± standard deviation of triplicate determination. Mean values in the same row followed by different letters are significantly (*p* < 0.05) different, BAMOnut: Bambara groundnut-*Moringa oleifera* snack bar, OB3: BAMOnut snack bar enriched with oats and 3% BAMOLP, MB2: BAMOnut snack bar enriched with millet and 2% BAMOLP, OMB5: BAMOnut snack bar enriched with oats, millet, and 5% BAMOLP, RUTF: Ready-to-use therapeutic food.

## Data Availability

No additional data were generated other than those reported in the manuscript.
